# Recent advances in understanding contextual TGFβ signaling

**DOI:** 10.12688/f1000research.11295.1

**Published:** 2017-05-26

**Authors:** Arshad Ayyaz, Liliana Attisano, Jeffrey L Wrana

**Affiliations:** 1 Center for Systems Biology, Lunenfeld-Tanenbaum Research Institute, Mount Sinai Hospital, Toronto, ON, Canada; 2 Department of Biochemistry and Donnelly Centre, University of Toronto, Toronto, ON, Canada; 3 Department of Molecular Genetics, University of Toronto, Toronto, ON, Canada

**Keywords:** TGFβ signalling, stem cells, cancer, cancer-associated stroma, hippo pathway, glioma, hematopoiesis, immunity.

## Abstract

The appearance of the first animal species on earth coincides with the emergence of transforming growth factor β (TGFβ) pathways. The evolution of these animals into more complex organisms coincides with a progressively increased TGFβ repertoire through gene duplications and divergence, making secreted TGFβ molecules the largest family of morphogenetic proteins in humans. It is therefore not surprising that TGFβ pathways govern numerous aspects of human biology from early embryonic development to regeneration, hematopoiesis, neurogenesis, and immunity. Such heavy reliance on these pathways is reflected in the susceptibility to minor perturbations in pathway components that can lead to dysregulated signaling and a diverse range of human pathologies such as cancer, fibrosis, and developmental disorders. Attempts to comprehensively resolve these signaling cascades are complicated by the long-recognized paradoxical role the pathway plays in cell biology. Recently, several groups have probed examples of the disparate aspects of TGFβ biology in a variety of animal models and uncovered novel context-dependent regulatory mechanisms. Here, we briefly review recent advancements and discuss their overall impact in directing future TGFβ research.

## Introduction

**Figure 1.**
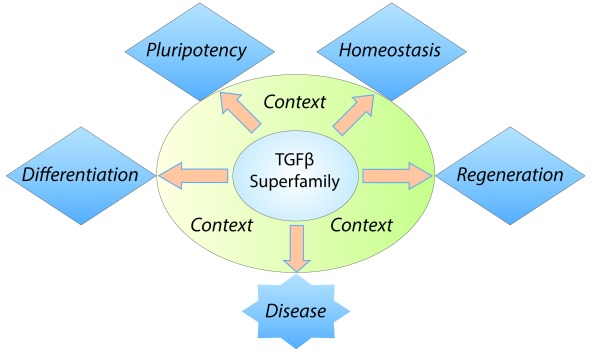
Context-dependent transforming growth factor β TGFβ superfamily signaling. Diverse biological activities regulated by TGFβ pathways are rigorously controlled in a finely calibrated contextual framework. Perturbation in these processes can lead to disease development.

Transforming growth factor β (TGFβ) signaling pathways have co-evolved with animals ^1^. Ligands of the TGFβ superfamily such as TGFβ(1–3), bone morphogenetic proteins (BMPs), activins, nodal, and growth differentiation factors (GDFs) control numerous aspects of animal biology including embryonic development, organogenesis, cell fate decisions, immune modulation, stress responses, and stem cell function ^2– 7^. Owing to widespread functional diversity, disruptions in TGFβ signaling are also associated with human diseases including cancer, fibrosis, systemic sclerosis, and hereditary disorders ^8– 12^. TGFβ morphogens often drive contradictory biological outcomes ( Figure 1), which has perplexed investigators for decades ^2, 9, 13– 15^. For example, TGFβ1, first described as a transforming growth factor, is also a potent growth inhibitor at benign stages of cancer and promotes metastasis, genetic heterogeneity, and drug resistance in aggressive carcinomas ^8, 9, 13, 14^. Similarly, BMPs also have diverse functions. BMPs prevent self-renewal of the intestinal stem cells (ISCs) by inhibiting Wingless-type MMTV integration site (WNT)/β-catenin signaling, yet promote specification, expansion, and homing of hematopoietic progenitors ^16– 21^. Although multi-layer regulation of TGFβ signaling can theoretically explain the dichotomy of activities, the exact molecular contexts that lead to opposite biological output in intact tissues remain elusive. Recent studies utilizing multiple animal and human models have addressed these contradictions and uncovered complex interactions in immune and regenerative tissues that determine specific biological outcomes of TGFβ activity. This review will briefly summarize these findings.

## TGFβ signal transduction

Canonical TGFβ Smad signaling is fairly simple ^2, 6, 22^. Activated ligands, released by the cleavage of their pro-domains in the extracellular matrix, bind to a complex of two type II and type I transmembrane receptors, inducing type II-mediated phosphorylation of type I receptors at specific serine/threonine residues. This in turn triggers the activation of regulatory Smad (R-Smad) transcription factors, which then complex with the co-transcription factor Smad4 and accumulate in the nucleus, where they alter gene expression ^2, 6, 22^. Inhibitory Smads (I-Smads), which are themselves induced by TGFβ family signaling ^23^, function in a negative feedback loop to regulate TGFβ signaling. The mammalian genome encodes 33 ligands, five type II and seven type I receptors, and eight Smad proteins (including five R-Smads, two I-Smads, and a single co-Smad, Smad4). In *Drosophila*, there are seven ligands, three type II receptors, two type I receptors, two R-Smads (Mad and dSmad2, called Smox), one co-Smad (Medea or Med), and one I-Smad (Dad) ^6, 22, 24, 25^. TGFβ signaling is further divided into discrete pathways, as functionally redundant TGFβ ligands generally activate a restricted set of corresponding receptors and Smad transcription factors. For example, TGFβ(1–3) activate Smad2/3 via TGFβ receptors, BMPs stimulate Smad1/5/8 activation via BMP or activin receptors, and activins, inhibins, and GDFs activate activin receptors to phosphorylate Smad2/3 ^2, 6, 22^. However, there are exceptions to this general model, as ligand/receptor promiscuity can lead to crosstalk between these distinct pathways ^26, 27^.

## TGFβ signaling in intestinal repair and colon cancer

Tissue homeostasis is maintained by a rapidly cycling population of LGR5 ^+^ crypt base columnar ISCs that reside at the bottom of crypts of Lieberkühn ^28^. As ISCs divide asymmetrically, their progeny expands in the transit-amplifying compartment and differentiates into functional lineages as they migrate towards the tips of villi. This baso-apical structural morphology in the mammalian intestine is maintained by two opposing concentration gradients of WNT and BMP signaling pathways ^29^ that sustain the basal regenerative stem/progenitor cell zone and apical differentiated zone, respectively. Indeed, in humans, inactivating mutations in adenomatous polyposis coli (APC) as well as the BMP receptor 1A (BMPR1A) in ISCs lead to hyperactivate β-catenin function and loss of BMP signaling, respectively, thus enhancing stem cell features and predisposing mutation carriers to cancer development ^30, 31^. TGFβ also promotes differentiation in the apical regions, and inactivating mutations in core TGFβ components are frequently detected in colorectal cancer (CRC). Intriguingly, recent studies propose that the activity of the canonical TGFβ pathway is enhanced by the non-canonical WNT ligand Wnt5A during acute injury in the intestine, and this promotes *de novo* generation and fission of crypts that facilitate efficient repair of the intestine ^32, 33^. At the molecular level, since the Hippo signaling pathway co-transcription factor yes-associated protein (YAP) is also required for crypt regeneration ^34^, interaction between TGFβ and Hippo pathways may explain these observations (see below).

## TGFβ signaling in immune regulation

The intestinal epithelium is inhabited by trillions of microbes that are kept in check by diverse epithelial and cellular immune responses ^35– 37^. The activity of the immune system, however, has to be tightly controlled to prevent inflammatory disorders and maintain homeostatic regeneration of tissues. TGFβ signaling is involved in the regulation of these processes ^38– 43^. Previously, TGFβ signaling was shown to prevent inflammation of the bowel upon activation in the associated dendritic cells ^44^. New data shed further light on this process by suggesting that *Clostridium* species within the microbiota induce TGFβ1 expression in colonic dendritic cells in a model of experimental colitis ^45^. This in turn stimulates the generation of immunosuppressive regulatory T (Treg) cells, thereby ameliorating the bowel colitis. TGFβ1 also amplifies its own production by activating a Smad3-dependent autocrine signaling loop within dendritic cells, which is, surprisingly, inhibited by Smad2 activity ^45^. Although Smad2 regulates TGFβ receptor expression in this context, it is not clear what drives the opposing roles of the closely related Smad3 and Smad2.

**Figure 2.**
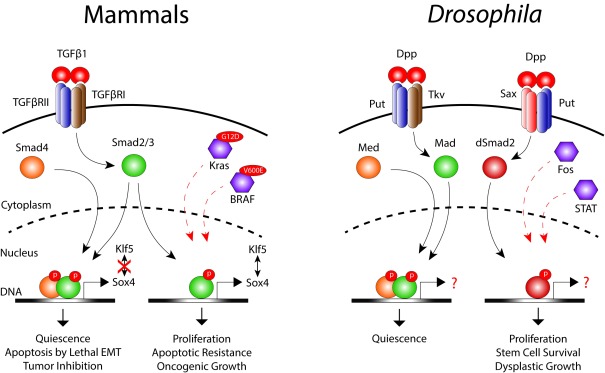
Transforming growth factor β TGFβ signaling during homeostasis and cancer. In mammals, TGFβ1 activates TGFβ receptors to recruit the canonical Smad2/3–Smad4 complex that, upon nuclear translocation, promotes quiescence and induces lethal epithelial-mesenchymal transition (EMT) to limit tumor growth. Loss of Smad4 in Kras ^G12D^-mutant cells as well as acquisition of a BRAF ^V600E^ mutation divert the activity of phosphorylated Smad2/3 to promote cell proliferation, survival, and metastatic growth. Similarly, *Drosophila* Decapentaplegic (Dpp) stimulates Punt (Put) and Thickveins (Tkv) receptors to activate the canonical Mad-Med (Smad4 homologue) nuclear complex that promotes quiescence. Overactivation of epidermal growth factor receptor (EGFR)/Fos and Jak/signal transducer and activator of transcription (STAT) signaling re-directs Dpp to activate the alternate Put–Saxophone (Sax) receptor complex. This complex then activates dSmad2, which translocates into the nucleus independently of Med and induces tumor-like stem cell growth and dysplasia.

TGFβ signaling was also found to mediate immune-stem cell communication during intestinal repair in *Drosophila*. Macrophages are recruited to the intestinal epithelium upon injury where they secrete the TGFβ/BMP homologue Decapentaplegic (Dpp) ^42, 46^. Dpp then activates the type II receptor Punt (Put) and type I receptor Saxophone (Sax) complex in the ISCs, which drives nuclear localization of dSmad2, thereby triggering ISC proliferation. This immune regulation of ISC function is required for efficient repair of the damaged intestine and is paramount for survival during pathogen invasion. Interestingly, ISCs can subsequently change their response to Dpp by expressing another type I receptor, Thickveins (Tkv), which, upon activation, phosphorylates the R-Smad Mad to restore stem cell quiescence. Oncogenic transformation of ISCs induced by ectopic expression of epidermal growth factor receptor (EGFR) and Jak/STAT pathways can supersede the growth inhibitory effects of Tkv/Mad signaling and requires chronic dSmad2 activity to promote tumor-like stem cell growth and hyperplasia ^46^ ( Figure 2). Finally, macrophage-derived Dpp also enhances age-related stem cell dysplasia and epithelial permeability through Sax/dSmad2 signaling ^46, 47^. Thus, TGFβ signaling has emerged as an important modulator of immune responses during tissue injury, regeneration, and aging.

## TGFβ signaling in tumor-associated stroma

TGFβ, BMP, and activin signaling play important roles in the crosstalk between tumor cells and the surrounding stroma, although the precise mechanisms are poorly understood ^48, 49^. Interestingly, poor prognosis in CRC patients with a stem/serrated/mesenchymal (SSM) subtype was recently linked to the activation of TGFβ signaling within cancer-associated fibroblasts (CAFs), which increased the frequency of tumor-initiating cells and the promotion of metastatic invasion in humanized mouse models ^50, 51^. Parallel observations were also made in breast cancer patients where both heat shock factor 1 (HSF1) and TGFβ signaling activation in CAFs is associated with poor clinical outcome ^52^. Another study showed that TGFβ signaling induces a mesenchymal transcriptional footprint in genetically engineered intestinal organoid cultures carrying a BRAF ^V600E^ mutation ^53^ ( Figure 2). Considering the heterogeneous nature of CRC and the paradoxical roles of TGFβ in stem cell regulation and CRC progression, continued analyses of interactions between mesenchymal and intestinal tissues under normal and dysplastic conditions are required.

## A role for Smad4 in switching TGFβ signaling in cancer cells

High TGFβ concentrations are associated with drug resistance and poor prognosis in gastrointestinal cancer patients, yet inactivating mutations in core TGFβ components are frequently found in these maladies. Indeed, deletion of Smad4 in normal intestinal tissues in combination with mutations in APC, TP53, and Kras lead to the formation of invasive carcinoma in mouse xenografts ^54^. Although the immunosuppressive activities of TGFβ1 in the tumor microenvironment have been accepted as one rational explanation, this does not explain the role that TGFβ1 plays within tumor cells lacking functional Smad4 to promote proliferation, metastasis, and apoptotic resistance ^9, 13, 14^. However, in pancreatic cancer, David *et al*. ^55^ have now identified Smad4 as a switch between tumor-suppressive and tumor-promoting activities of TGFβ in the tumor cells. Mechanistically, they show that TGFβ induces the expression of Sox4 in a Smad4-independent manner that then cooperates with the epithelial lineage determinant Kruppel like factor 5 (Klf5) to promote oncogenic growth in Kras ^G12D^-driven cancers. Restoration of Smad4 activity disrupts Sox4–KLF5 interaction and redirects Sox4 to induce the transcription of pro-apoptotic genes through a lethal epithelial-mesenchymal transition (EMT) program ^55^ ( Figure 2). Thus, Smad4 functions as a transcriptional switch empowering TGFβ1 to control two different transcriptional programs, each leading to opposite outputs. Interestingly, these findings are in agreement with fly studies where dSmad2 was found to promote tumor-like ISC growth and hyperplasia in a Smad4-independent manner ^46^.

## Molecular mechanisms underlying contextual TGFβ signaling

The Hippo pathway plays a critical role in cell and organ growth and is implicated in tissue regeneration and cancer ^34, 56– 58^. In the Hippo pathway, a core kinase cassette comprising MST1/2 and LATS1/2 kinases phosphorylate and thereby sequester the related transcriptional co-activators YAP and transcriptional co-activator with PDZ-binding motif (TAZ) in the cytoplasm, which can also lead to their degradation. Upon inhibition of MST/LATS kinase cassette, YAP/TAZ translocate into the nucleus, where they associate with the DNA-binding partners, such as the TEA domain transcription factors (TEADs), to regulate gene expression. The Hippo pathway is an environmental sensor that responds to a wide range of extrinsic cues that include cell density, cell polarity, metabolic cues, GPCR signaling, and mechanotransduction. The Hippo pathway in turn interacts with several signaling cascades including TGFβ, WNT, EGFR/mitogen-associated protein kinase (MAPK), and others to regulate cell proliferation, tissue growth, apoptotic resistance, and tumor progression ^56– 58^. In the case of TGFβ signaling, when the Hippo pathway is inactive, YAP/TAZ physically interact with the Smad2/3–Smad4 complex to promote its nuclear accumulation, thereby regulating diverse transcriptional programs that maintain pluripotency in embryonic stem cells ^59– 61^, regulate differentiation into diverse lineages ^62– 67^, enhance wound healing ^68^, and promote tumorigenesis in breast cancer, mesothelioma, and hepatocarcinoma models ^69– 71^. Recent studies have also uncovered cooperative interactions between YAP and TGFβ-regulated Smads that couple mechanotransduction in fibroblasts to TGFβ-induced profibrotic gene expression programs that are important in fibrosis ^72, 73^. Interestingly, the TGFβ pathway can also regulate Hippo signaling by interacting with the scaffold protein RASSF1A, which is often inactivated in human cancers ^74^. Mechanistically, high TGFβ activity promotes ubiquitination-based degradation of RASSF1A. This inactivates the MST/LATS kinase cassette, facilitating nuclear localization of the YAP/Smad complex, thereby enhancing the tumorigenic potential of TGFβ signaling. Together, these studies indicate that the TGFβ and Hippo pathway interactions are finely tuned and disruptions in these processes can alter stem cell function and cell fate decisions and lead to fibrosis and cancer.

## TGFβ pathway and hematopoiesis in mammals

TGFβ pathways control several aspects of blood formation ^21, 75^. BMPs have been shown to specify hematopoietic tissues during development and promote heterogeneity by differentially regulating the formation of diverse hematopoietic stem cell (HSC) types that are separately deposited in the fetal liver and bone marrow of developing embryos ^20, 21, 76^. Moreover, BMPs modulate myeloid lineage specification and promote homing and proliferation of hematopoietic progenitors at postnatal stages ^18, 21, 77^. TGFβ(1–3) ligands, however, are potent inhibitors of hematopoiesis in adults ^21, 78^. New studies now identify that megakaryocytes secrete the bulk of latent TGFβ1 in the bone marrow, which is cleaved by the non-myelinating Schwann cells ^79, 80^. Once activated, TGFβ1 stimulates canonical Smad2/3 signaling in multipotent HSCs to promote quiescence. Intriguingly, megakaryocyte-derived TGFβ1 also protects HSCs from exhaustion in a hostile microenvironment, which could be relevant to the development of hematological malignancies ^79^. Indeed, leukemia-initiating cells (LICs) in chronic myeloid leukemia (CML) derive therapeutic resistance from active TGFβ signaling ^81^. Although a direct link between megakaryocytes/platelets and the development of hematological malignancies has not been established, platelets are shown to activate TGFβ signaling in circulating tumor cells, enhance immune evasion, and facilitate bone metastasis ^82, 83^.

## BMP signaling in glioma

Gliomas are the most common tumors of the central nervous system ^84, 85^. Among these, diffuse intrinsic pontine gliomas (DIPGs) almost exclusively occur in children at the age of 6–8 years. DIPGs exhibit a dismal prognosis with a median survival rate of only 1 year ^86^ because no therapeutic treatment is effective against these cancers, primarily owing to their anatomical location. Whole genome exploration studies have identified unique epigenetic changes in DIPG specimens with 78% of patients exhibiting a single amino acid mutation in a histone H3 variant, a lysine replacement to methionine at position 27 (K27M). This histone H3-K27M mutation removes trimethylation from the histone H3 tail, allowing transcriptional derepression that is associated with the induction of stem cell features and cancer development ^87^. Intriguingly, four independent studies have now shown that a majority of patients who have the K27M mutation also bear activating mutations in the activin receptor type 1 (ACVR1) ^88– 92^. Activity of mutant ACVR1 still requires ligand-mediated activation of type II receptors and does not seem to be tumorigenic alone, since the same ACVR1-activating mutations found in hereditary fibrodysplasia ossificans progressiva (FOP) do not result in cancer predisposition ^93, 94^. ACVR1 has not been previously implicated in cancer, thus the link with a mutated histone H3 variant in the context of glioma is particularly intriguing.

## Conclusions and perspectives

Extensive efforts in TGFβ research have revealed that dynamic interactions between tissues and rapidly changing molecular contexts determine the specific biological output of TGFβ signaling in intact tissues. These complex processes are designed to fine-tune cellular reactions and prevent exaggerated responses that may lead to lethal consequences in animals. These interactions also allow TGFβ pathways to sense a wide spectrum of constantly changing cellular microenvironments and launch appropriate responses. This complex pattern of tight TGFβ regulation also enables just a few components to control diverse biological activities and creates opportunities for diseased cells to adapt and modify these elements to use for their own growth and survival. Therefore, while it is necessary to keep an open mind when considering such complexities in TGFβ signaling, caution must be exercised when using current tools and interpreting specific observations. This is particularly true in pathological tissues where canonical signaling pathways may be altered. In future work, it will be vital to incorporate information about TGFβ pathway crosstalk and couple observations of pathway activity to mechanistic molecular and functional assays.

## Abbreviations

ACVR1, activin receptor type 1; APC, adenomatous polyposis coli; BMP, bone morphogenetic protein; CAF, cancer-associated fibroblast; CRC, colorectal cancer; DIPG, diffuse intrinsic pontine glioma; Dpp, Decapentaplegic; EGFR, epidermal growth factor receptor; GDF, growth differentiation factors; HSC, hematopoietic stem cell; ISC, intestinal stem cell; I-Smad, inhibitory Smad; K27M, lysine replacement to methionine at position 27; Klf5, Kruppel like factor 5; R-Smad, regulatory Smad; Sax, Saxophone; TAZ, transcriptional co-activator with PDZ-binding motif; TGFβ, transforming growth factor beta; Tkv, Thickveins; WNT, Wingless-type MMTV integration site; YAP, yes-associated protein.
